# Cognitive Control Over Visual Motion Processing – Are Children With ADHD Especially Compromised? A Pilot Study of Flanker Task Event-Related Potentials

**DOI:** 10.3389/fnhum.2018.00491

**Published:** 2018-12-05

**Authors:** Bettina Lange-Malecki, Stefan Treue, Aribert Rothenberger, Björn Albrecht

**Affiliations:** ^1^German Primate Center – Leibniz Institute for Primate Research, Göttingen, Germany; ^2^Leibniz-ScienceCampus Primate Cognition, Göttingen, Germany; ^3^Bernstein Center for Computational Neuroscience, Göttingen, Germany; ^4^Faculty for Biology and Psychology, University of Göttingen, Göttingen, Germany; ^5^Department of Child and Adolescent Psychiatry and Psychotherapy, University Medical Center Göttingen, Göttingen, Germany

**Keywords:** cognitive control, visual motion, Flanker-Task, N2, error negativity (Ne ERN), error positivity (Pe), attention deficit/hyperactivity disorder (ADHD)

## Abstract

Performance deficits and diminished brain activity during cognitive control and error processing are frequently reported in attention deficit/hyperactivity disorder (ADHD), indicating a “top-down” deficit in executive attention. So far, these findings are almost exclusively based on the processing of static visual forms, neglecting the importance of visual motion processing in everyday life as well as important attentional and neuroanatomical differences between processing static forms and visual motion. For the current study, we contrasted performance and electrophysiological parameters associated with cognitive control from two Flanker-Tasks using static stimuli and moving random dot patterns. Behavioral data and event-related potentials were recorded from 16 boys with ADHD (combined type) and 26 controls (aged 8–15 years). The ADHD group showed less accuracy especially for moving stimuli, and prolonged response times for both stimulus types. Analyses of electrophysiological parameters of cognitive control revealed trends for diminished N2-enhancements and smaller error-negativities (indicating medium effect sizes), and we detected significantly lower error positivities (large effect sizes) compared to controls, similarly for both static and moving stimuli. Taken together, the study supports evidence that motion processing is not fully developed in childhood and that the cognitive control deficit in ADHD is of higher order and independent of stimulus type.

## Introduction

Attention Deficit/Hyperactivity Disorder (ADHD) is a common early-onset neurodevelopmental disorder, characterized by severe and age-inappropriate levels of pervasive inattention, hyperactivity and impulsivity that occurs in about 5% of school-aged children with a strong overrepresentation of boys (APA, 2013; Polanczyk et al., 2015). ADHD is regarded in many ways as a heterogeneous disorder, and symptoms may be consequences of motivational, cognitive or self-regulation deficits associated with distinct brain dysfunctions (Sagvolden et al., 2005; Sergeant, 2005; Sonuga-Barke, 2005). The current study dwells on cognitive and self-regulation difficulties, probably associated with dysfunctions in fronto-striatal dopaminergic networks that lead to deficits in executive functions in general and cognitive control in particular (Pennington and Ozonoff, 1996; Barkley, 1997; Sergeant, 2005; Sonuga-Barke, 2005; Cortese et al., 2012).

Cognitive control comes into play when task demands or performance errors require rapid adaptation. This can be tapped by the Eriksen Flanker Task, which is a demanding paradigm that requires responding to a target stimulus in the presence of competing distractors (Eriksen and Eriksen, 1974). Children with ADHD typically display several performance deficits during such tasks, e.g., their responses tend to be slower, more variable and more error-prone (Mullane et al., 2009).

The associated brain electrical activity underlying those task demands, performance difficulties and hence potential deficits in patients can be tracked with high temporal resolution using Electroencephalography (EEG) and thereof extracted Event-Related Potentials (ERP) (Banaschewski and Brandeis, 2007). Task demands may be reflected in the stimulus-locked ERP. As an example, the aforementioned Flanker-Task elicits a fronto-central negativity, peaking around 200–400 ms after onset of the stimulus (N2) which is larger when the target is primed with incongruent compared to congruent distractors, suggesting that this N2-Enhancement is driven by elevated cognitive control tapped when monitoring stimulus conflict (Donkers and Van Boxtel, 2004; Falkenstein, 2006). Sources of N2 have been localized in the anterior cingulate cortex (ACC) as part of the fronto-striatal brain networks probably implicated in ADHD (Van Veen and Carter, 2002; Sonuga-Barke, 2005).

Cognitive control may be also implicated after performance errors that require adapting response strategies. In the response-locked ERP, errors are generally accompanied by a negative component peaking approximately 40–120 ms after the erroneous response at fronto-central sites (error negativity, Ne or error related negativity, ERN) with sources in the anterior cingulate cortex. Several theories ascribe Ne a crucial role in response monitoring, error detection or reinforcement learning (Falkenstein et al., 1990; Gehring et al., 1993; Holroyd and Coles, 2002), and some studies suggest that the stimulus-locked N2(-enhancement) and the response-locked Ne (with increased amplitude compared to the Nc evoked by correct responses) reflect a similar cognitive control process that is triggered by, respectively, different aspects of task performance (Ridderinkhof et al., 2002; Van Veen and Carter, 2002; Yeung and Cohen, 2006). The Ne is frequently followed by a more parietal positive deflection (error positivity, Pe) within 200–500 ms after the response (Gehring et al., 1993; Falkenstein et al., 2000). One of its characteristics is that it is elicited unlike Ne only after full errors of which the subject is aware (Nieuwenhuis et al., 2001). Dipole modeling found a generator of Pe in the rostral ACC, which suggests that it may reflect affective error assessment (Van Veen and Carter, 2002).

Numerous studies and several meta-analyses suggest that these electrophysiological parameters of cognitive control (N2) and error processing (error negativity, Ne or ERN as well as the later error positivity, Pe) may be diminished in ADHD (Shiels and Hawk, 2010; Geburek et al., 2013; Johnstone et al., 2013). Moreover, a recent study with patients revealed that deficits in performance and brain electrical activity after errors (Ne, Pe) can be ameliorated by incentives and methylphenidate as frequently used intervention options in ADHD (Groom et al., 2013).

Most ADHD studies of cognition in general and cognitive control in particular have used static visual stimuli such as letters or forms [triangles or symbols, e.g., by (Jonkman et al., 1999; Albrecht et al., 2008; McLoughlin et al., 2009; Mullane et al., 2009; Michelini et al., 2016)]. This focus on aspects of visual form means that the corresponding tasks rely on sensory information processing along the ventral pathway in visual cortex, a hierarchical chain of cortical areas specialized for the identification of visual stimuli, based on their color, orientation and other form aspects (Ungerleider and Mishkin, 1982; Kravitz et al., 2013). The historical focus on the ventral pathway means that very little is known about cognitive control during processing of moving visual stimuli in ADHD, despite the prime importance of visual motion perception for everyday life, such as appropriate visually guided behavior as well as the frequent clinical observation that children with ADHD are easily distracted by movements in their environment.

This ventral “what” processing stream is paralleled by a dorsal “where” pathway of cortical areas specialized for the spatial layout of the visual environment and visual movement, thought to support accurate visually guided movements (Ungerleider and Mishkin, 1982; Goodale and Milner, 1992; Kravitz et al., 2011). Moving stimuli are able to capture automatic attention, not only under the condition of abrupt appearance of a new object (see, e.g., Yantis and Jonides, 1990) or salient discontinuities (Kawahara et al., 2012), but also when they contain translating and looming motion, whereas receding stimuli do not attract automatic attention (Franconeri and Simons, 2003). Psychophysically, human motion processing is substantially affected by the voluntary use or ignorance of the motion information to a task (Raymond, 2000; Katzner et al., 2006; Tzvetanov et al., 2006).

The central question of our study is whether children with ADHD display specific problems with motion processing during cognitive control versus processing of static forms. Although beyond the objectives of our experiment, this may well be a consequence of difficulties in the magnocellular pathway and amongst others in functioning of the superior colliculus, which play a role in visual motion processing as well as voluntary attention shifts, that may be implicated in several difficulties observed in ADHD (Overton, 2008). In the current study, we contrast performance and electrophysiological parameters associated with cognitive control obtained from visual Flanker-Tasks with static forms and moving random dot patterns (RDPs) in children with ADHD compared to Controls. In a previous study with healthy Controls, both stimuli yielded comparable performance and congruency effects (Lange-Malecki and Treue, 2012). We hypothesize in trials with incongruent stimuli more errors, slower correct responses and enhanced N2 amplitude in both static arrowhead and motion Flanker-Tasks. Because of the automatic capturing of attention during motion processing, we expect that these congruency effects will be larger for RDPs compared to static arrowheads. Consequently, we expect diminished performance, lower N2-enhancement and diminished Ne or Pe in children with ADHD particularly when processing motion.

However, especially children with ADHD have been associated with hypoarousal at rest and activation difficulties during task performance that may partly explain impaired performance in terms of slower and more variable responses in a variety of demands (Sergeant, 2005). This has been demonstrated by manipulating state regulation factors during task performance [e.g., event-rate, see Uebel et al. (2010)]. Arousal and activation are associated with vagal tone as reflected in skin conductance level, and studies in children indicate also close relation with brain electrical alpha activity (Barry et al., 2009a,b). For testing the auxiliary hypothesis that processing of visual motion may lead to elevated activation that may be especially beneficial in ADHD, we assessed absolute Alpha power during Task performance, which is expected to be inversely related to arousal and activation level.

## Materials and Methods

This study was carried out in accordance with the recommendations of the ethics committee of the University Medical Center Göttingen with written informed consent from all subjects (including all children, because they were 8 years and older, and thus able to do so). All subjects gave written informed consent in accordance with the Declaration of Helsinki. The protocol was approved by the ethics committee of the University Medical Center Göttingen.

### Sample

A total of 20 boys with ADHD and 31 male controls, aged 8–15 years, participated in the study. Children in the ADHD group were patients of the Clinic for Child and Adolescent Psychiatry and Psychotherapy at the University of Göttingen or outpatients of a nearby board-certified private practice. The control group was recruited after information talks given in schools in the region of Göttingen. Detailed information sheets about the study were provided to parents and children, and written informed consent was obtained from both. Children taking stimulants were off medication for at least 48 h before testing. All children received small prizes and a financial compensation (15 €) for participation.

All subjects had normal or corrected-to-normal vision, an full-scale IQ above 85 [estimated from the WISC sub-tests Picture Completion, Similarities, Block Design and Vocabulary according to (Sattler, 1992)] and no childhood psychiatric disorders that might mimic ADHD (e.g., Autism Spectrum Disorder) except co-existing oppositional defiant/conduct disorder or learning difficulties (ICD-10 F80.x, F81.x, and F83.0). ADHD was diagnosed by a clinical assessment according to ICD-10 criteria of hyperkinetic disorder (F90.0) or hyperkinetic conduct disorder (F90.1) with extended age of onset, which were, respectively, in accordance with DSM criteria of ADHD combined type (APA, 1994, 2013). Further clinical questionnaires were used for both groups as screening instruments to detect more general mental health problems: Child- and Behavior Check List for parents (CBCL) and Teachers Report Form (TRF) plus two rating scales about ADHD (FBB-HKS) and oppositional defiant and conduct disorder (FBB-SSV) according to ICD-10 and DSM-IV (Doepfner and Lehmkuhl, 2000; Achenbach et al., 2008). In addition, the Strength- and Difficulties Questionnaires (SDQ) for parents and teachers which addresses more general clinically relevant aspects was used for characterizing the psychopathological profile of Controls and ADHD patient groups (Goodman, 1997; Woerner et al., 2004).

Due to too few correct responses, too many performance errors (more than 50% errors in the congruent condition) or artifacts in the EEG leading to less than 20 sweeps in the respective ERP, 9 datasets (4 ADHD, 5 controls) had to be excluded, but the exclusion ratio did not differ between groups [χ^2^_(1)_ = 0.13, *p* = 0.72]. The included samples of 16 children with ADHD and 26 controls were age-matched [*F*_(1,40)_ = 0.08, *p* = 0.8, $\eta_{p}^{2}$< 0.01] and did not differ concerning age distribution. Prorated IQ was slightly higher in the control group [*F*_(1,40)_ = 3.9, *p* = 0.05, $\eta_{p}^{2}$= 0.09]. Psychopathological ratings with the SDQ from parents and teachers revealed difficulties in children with ADHD regarding Hyperactivity, Conduct Problems, Peer Problems and Total Problems scores (all *F* > 7.7, *p* < 0.01, see Table 1). In particular, considering the SDQ parents Hyperactivity/Impulsivity rating norms (Woerner et al., 2004), all but one Control participant scored within normal range, whilst in the ADHD group 80% of the patients showed abnormal scores.

**Table 1:** Sample description.

	Controls (*N* = 26)	ADHS (*N* = 16)	ANOVA		
	Mean (*SD*)	Mean (*SD*)	*F*_(1,40)_	*p*	$\eta_{p}^{2}$
**Age** (in months)	133.0 (18.9)	131.1 (22.6)	0.1	–	0.01
**IQ** (estimated)	115.0 (12.1)	107.2 (12.5)	**3.9**	**+**	**0.09**
**SDQ parents**^a^					
Emotional symptoms	0.9 (1.0)	2.9 (2.7)	**12.0**	**^∗∗^**	**0.24**
Conduct problems	1.0 (1.0)	3.5 (2.3)	**23.1**	**^∗∗^**	**0.37**
Hyperactivity	1.7 (1.8)	7.5 (1.8)	**103.4**	**^∗∗^**	**0.73**
Peer problems	0.8 (0.9)	2.2 (1.6)	**13.8**	**^∗∗^**	**0.26**
Pro-social behavior	7.8 (1.9)	6.5 (1.6)	**5.1**	**^∗^**	**0.12**
Total problems	4.4 (2.9)	16.1 (5.5)	**82.4**	**^∗∗^**	**0.68**
Impact	0.2 (0.6)	2.9 (1.8)	**50.7**	**^∗∗^**	**0.57**
**SDQ teachers**^b^					
Emotional symptoms	0.3 (0.8)	0.8 (1.0)	2.3	–	0.07
Conduct problems	0.6 (0.9)	2.3 (2.5)	**7.7**	**^∗∗^**	**0.2**
Hyperactivity	1.1 (1.5)	7.1 (3.1)	**55.0**	**^∗∗^**	**0.65**
Peer problems	0.4 (0.7)	3.4 (2.5)	**25.5**	**^∗∗^**	**0.46**
Pro-social behavior	7.5 (2.6)	5.8 (2.2)	**3.3^-^**	**+**	**0.10**
Total problems	2.3 (3.0)	13.6 (7.3)	**38.8**	**^∗∗^**	**0.56**
Impact	0.1 (0.3)	2.3 (1.5)	**42.8**	**^∗∗^**	**0.59**

*Sample description of 26 control boys and 16 boys with ADHD for five characteristic behavioral symptoms for ADHD from Strength and Difficulties Questionnaires (SDQ). Mean age and calculated IQ were exhibited for both groups. Analysis of variance (ANOVA) has been performed with degrees of freedom (F), significances (p) and effect strengths ($\eta_{p}^{2}$). ^*a*^SDQ Parents not available for one subject, df = 1;39, ^*b*^SDQ Teachers not available for 10 subjects, df = 1;30, significance denoted by ^∗∗^p < 0.01, ^∗^p < 0.05, +p < 0.1*. Bold lines indicate at least medium effects with at least *p* < 0.10.

### Stimuli and Task

**FIGURE 1:**
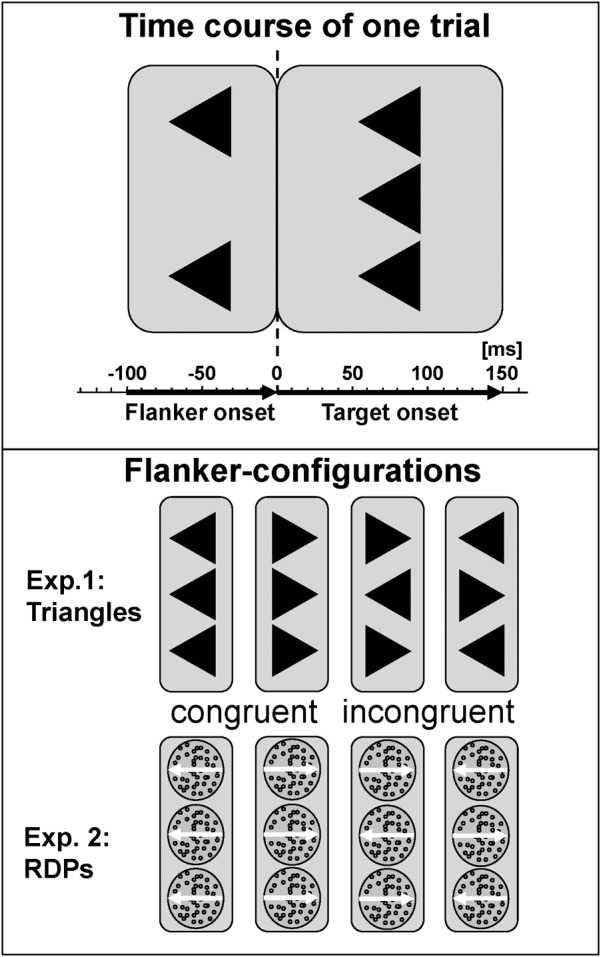
Task description. Flankers preceded presentation of the central target by 100 ms, which persists together with the flankers for a further 150 ms. Stimuli were static arrowheads or dynamic RDPs, conditions were congruent or incongruent, and responses were required to the left or right. Stimulus-onset asynchrony was 1650 ms, respectively. Bold lines indicate at least medium effects with at least *p* < 0.10.

Cognitive control during processing of static visual forms was tested with a classical arrowhead version of the Eriksen Flanker-Task (Eriksen and Eriksen, 1974; Kopp et al., 1996) in noise-shielded and slightly dimmed rooms. Each trial started with the presentation of the centrally located fixation cross. First, only the two flankers appeared for 100 ms to prime response competition, followed by adding the target to the flankers for a further 150 ms. This 100 ms delay is the maximum in the inverted-U-shape relation between flanker-to-target onset asynchrony and magnitude of the flanker-effect for static arrowheads (Mattler, 2003). For testing visual motion, we modified this configuration for the use of RDP with small black dots moving coherently either left- or rightwards as target and flankers, arranged vertically in the same manner as the arrows (see Figure 1). Stimuli were presented in the center of a 17″ CRT monitor (resolution 800 × 600) on a light gray background at 90 cm viewing distance using Presentation 9.90 software from Neurobehavioral Systems. The edge length of equilateral arrowheads as well as the diameter of the circular RDPs was set to 1° with a distance between target and flanker of 1.25° from center to center. Dot density was 40/deg2, dot size 2 pixel × 2 pixel and dot speed 4°/sec. A trial was started every 1650 ms. These parameters were based on a previous study with healthy adults that revealed similar performance for static arrowheads and RDPs as well as clear congruency effects in reaction time and error rates (Lange-Malecki and Treue, 2012).

Subjects had to press response buttons with the index finger of the left or right hand corresponding to the target direction. On congruent trials, flanker and target pointed to the same, on incongruent trials to opposite, horizontal directions. Congruency (congruent vs. incongruent stimuli), and target direction (left vs. right), were balanced and randomized. Reaction times were measured with respect to the onset of the target.

Altogether, 400 trials were presented in ten blocks á 40 trials intermitted by written feedback displayed on screen for 8 s at the end of each block: if more than 10% errors on congruent or more than 40% errors on incongruent trials were committed, the child was instructed to be more accurate. If less than 10% errors in the congruent and less than 10% errors in incongruent trials occurred, the child was instructed to respond faster; otherwise, it was told to continue in the same way.

The duration of the Flanker-Tasks including two practice blocks of 24 trials was approximately 15 min each, and arrowhead and RDP Flanker-Tasks were presented in randomized order after 3 min of resting EEG with eyes open and –closed condition.

### Electrophysiological Recording and Processing

The electroencephalogram was recorded with Ag/AgCl electrodes from 23 sites according to the extended 10–20 System altogether with the electro-oculogram recorded from additional electrodes placed above and below the right eye and at the canthi with FCz as recording reference and a ground electrode placed at the forehead using a BrainAmp amplifier. Sampling rate was 500 Hz with low and high cutoff filters set to 0.016 and 100 Hz, respectively, and a 50 Hz notch filter. Impedances were kept below 10 kΩ.

Offline processing was performed using Brain Vision Analyzer software (Brain Products, Gilching, Germany). The EEG was downsampled to 256 Hz, re-referenced to the average and filtered offline with 0.1 – 15 Hz, 24 dB/oct Butterworth filters. Ocular artifacts were corrected using the method of Gratton and Coles without raw average subtraction (Gratton et al., 1983). If the amplitude at any EEG electrode exceeded ±100 μV, a section -100 to +800 ms was excluded from further analyses. Response locked (-500 ms to +1000 ms relative to button press) and stimulus-locked (-200 to +1825 ms around the target onset) segments were subsequently checked and averaged. The standard serial mouse, used to record responses, caused a response trigger delay of approximately 35 ms (standard deviation 2.2 ms; 2/3 of all responses were registered within ±2 ms around the mean latency of 35 ms), which was corrected for in the analyses (Chambers and Brown, 2003). To avoid distortion of ERP topography, no baseline subtraction was applied.

All averages contained at least 20 sweeps (more details can be found in the respective Tables of the Results). ERPs comprised more accepted sweeps in correct responses than errors and congruent vs. incongruent trials and also group-differences in numbers of sweeps (in general, ERPs from children with ADHD comprised fewer numbers of accepted sweeps, but only for response-locked errors they comprised more). Signal to noise ratio (SNR) was for response-locked ERPs higher in the condition with static arrowheads than moving RDPs, and also higher for correct responses than errors, and for stimulus-locked ERPs higher in ERPs from incongruent than congruent trials and particularly for incongruent trials ERPs highest at electrode Cz. However, there were no significant group differences or interaction effects involving Group (all *p* > 0.12, $\eta_{p}^{2}$ < 0.06).

For testing the hypothesis whether processing of moving RDPs may lead to elevated activation, we performed the preprocessing above except that the data was filtered offline 0.1 to 30 Hz/24 db/oct and conducted an Fast Fourier Transformation (FFT) with 10% Hanning window on 4 s non-overlapping artifact-free epochs to extract absolute Alpha (8–13 Hz) band power.

### Statistical Analyses

Analyses of performance data (RT and RT-SD from correct responses, error-rates) were conducted using a General Linear Model with between subjects factor ADHD (children with ADHD vs. Controls) and within subject factors Condition (static arrowhead vs. moving RDP Flanker-Task) and Congruency (congruent vs. incongruent trials). The electrophysiological parameters of cognitive control were also tested regarding factor “Electrode” (Fz, FCz, and Cz for the stimulus-locked N2). Error processing was tested with factors “ADHD,” “Error” (errors vs. correct responses in incongruent trials) and Electrode (only FCz for the Ne/Nc with fronto-central maximum, and Cz and Pz for the centro-parietal Pe/Pc). Alpha power was logarithmus naturalis (LN) transformed to achieve normal distribution and tested in an ANOVA with factors Group, Condition and Site (Fpz, Fz, Cz, Pz, and Oz).

The available sample size allows the detection of large between-subjects (ADHD-) effects (*d* ≤ 0.9 two-tailed) with a power of 1-β = 0.80 at the conventional significance level α = 0.05. Significant effects are indicated by ^∗∗^*p* < 0.01, ^∗^*p* < 0.05, and trends by +*p* < 0.1.

## Results

### Performance

**FIGURE 2:**
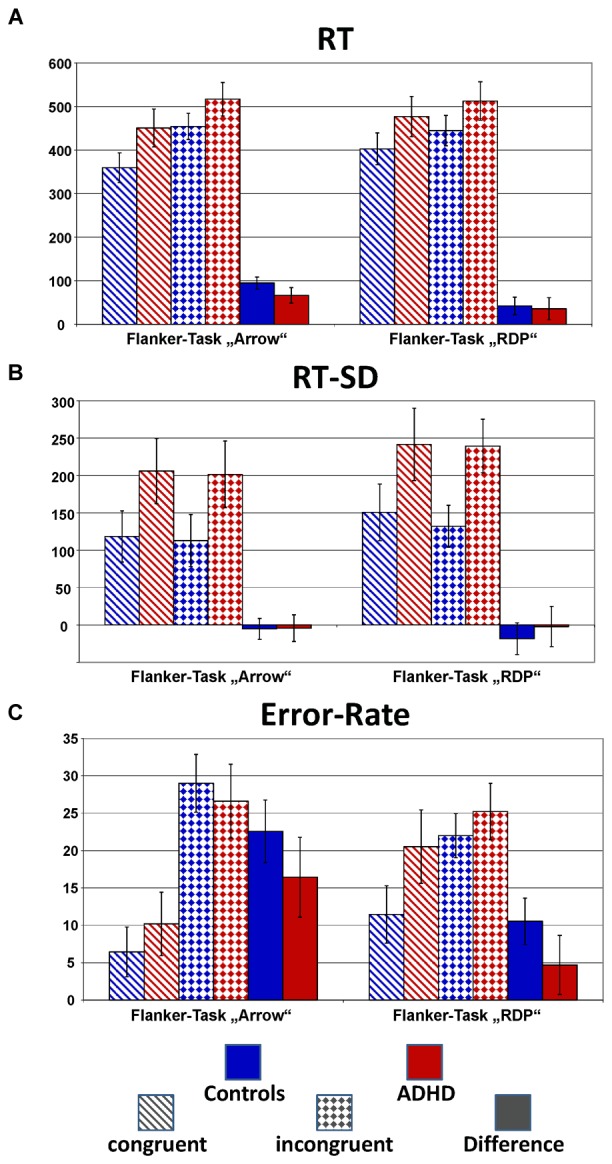
Performance data. This figure gives confidence intervals (*p* = 0.05) of reaction-times to correct responses (RT, **A**), intra-individual standard deviation of correct-response times (RT-SD, **B**) and error-rate **(C)** from Flanker-Tasks with static stimuli (“Arrow,” left) and RDPs (right), separately for congruent (dashed bars) and incongruent (checkered) stimuli as well as their difference (solid bars). Children with ADHD (red) showed slower and more variable responses than controls (blue), as well as higher error-rate already in the congruent condition.

Figure 2 plots the behavioral performance parameters from controls (blue) and children with ADHD (red) for the flanker tasks with static arrowheads (solid) and moving RDPs (checkered). Detailed statistical parameters are given in Table 2.

**Table 2:** Performance data of arrow flanker task and motion flanker task with statistics.

	Controls	ADHS	ANOVA			
	Mean (*SD*)	Mean (*SD*)	Test	*F*_(1,40)_	*p*	$\eta_{p}^{2}$
**Reaction times of correct responses (ms):**
**Arrow flanker task**			S	1.7	–	0.04
Congruent correct	359 (54)	451 (122)	**G**	**9.4**	**^∗∗^**	0.**19**
Incongruent correct	454 (65)	517 (93)	S × G	0.1	–	0.01
Difference	95 (25)	66 (47)	**C**	**125.4**	**^∗∗^**	**0.76**
**Motion flanker task**			C × G	2.7	–	0.06
Congruent correct	403 (76)	477 (113)	**S × C**	**23.0**	**^∗∗^**	**0.37**
Incongruent correct	445 (79)	512 (101)	S × C × G	1.6	–	0.04
Difference	42 (39)	36 (65)				
**RT-SD of correct responses (ms):**
**Arrow flanker task**			**S**	**13.3**	**^∗∗^**	**0.25**
Congruent correct	118 (57)	206 (121)	**G**	**14.2**	**^∗∗^**	**0.26**
Incongruent correct	113 (57)	202 (123)	S × G	0.4	–	0.01
**Motion flanker task**			C	1.8	–	0.04
Congruent correct	151 (80)	242 (118)	C × G	0.6	–	0.02
Incongruent correct	132 (49)	239 (97)	S × C	0.4	–	0.01
			S × C × G	0.7	–	0.02
**Error rate (%):**
**Arrow flanker task**						
Congruent correct	6 (9.3)	10 (6.6)	S	2.5	–	0.06
Incongruent correct	29 (10.5)	27 (8.5)	G	2.5	–	0.06
Difference	23 (11.0)	16 (9.9)	**S × G**	**6.1**	**^∗^**	**0.13**
**Motion flanker task**			**C**	**116.8**	**^∗∗^**	**0.75**
Congruent correct	11 (8.6)	21 (11.3)	**C × G**	**5.7**	**^∗^**	0.13
Incongruent correct	22 (6.9)	25 (8.3)	**S × C**	**57.8**	**^∗∗^**	0.59
Difference	11 (7.1)	5 (8.9)	S × C × G	0.1	–	0.01

****Left side:****Performance data of both groups for the arrow flanker task and the motion flanker task: mean reaction times of correct responses in ms, mean reaction time variability in ms and mean error rates in %, all with standard deviations, for congruent and incongruent designs and their differences (congruency effects).****Right side:****Statistics performed by analysis of variance (ANOVA): S, stimulus; G, group; C, congruency. ^∗∗^p < 0.01, ^∗^p < 0.05, +p < 0.1.* Bold lines indicate at least medium effects with at least *p* < 0.10.*

#### Mean Reaction Times of Correct Responses

Reaction times (RT, see Figure 2A) are significantly slower in the ADHD group [Group: *F*_(1,40)_ = 9.4, *p* < 0.01, $\eta_{p}^{2}$= 0.20]. Both groups show Congruency effects, i.e., incongruent items led to longer reaction times compared to congruent items for both tasks [Congruency: *F*_(1,40)_ = 125.4, *p* < 0.01, $\eta_{p}^{2}$= 0.76], which marginally differ between ADHD and Controls [Congruency × Group: *F*_(1,40)_ = 2.7, *p* = 0.11, $\eta_{p}^{2}$= 0.06, Stimulus × Congruency × Group: *F*_(1,40)_ = 1.6, *p* = 0.21, $\eta_{p}^{2}$= 0.04]. *Post hoc* comparisons of the Congruency effect revealed a significant group difference for static arrowheads (Figure 2A left panel: Controls: 95 ms and ADHD: 66 ms, *p* < 0.05), but not for RDPs (42 ms vs. 36 ms, respectively).

The intra-individual reaction time variability (RT-SD, Figure 2B) was higher for RDPs than arrowheads [Stimulus: *F*_(1,40)_ = 13.3, *p* < 0.01, $\eta_{p}^{2}$= 0.25], but did not differ in congruent from incongruent trials [Congruency: *F*_(1,40)_ = 1.8, *p* = 0.18, $\eta_{p}^{2}$ = 0.04]. Moreover, RT-SD was significantly larger in the ADHD group [Group: *F*_(1,40)_ = 14.2, *p* < 0.01, $\eta_{p}^{2}$ = 0.26] without any further interactions (see Table 2 and Figure 2B).

#### Mean Error-Rates

All children made significantly more errors in incongruent compared to congruent trials [Congruency: *F*_(1,40)_ = 116.8, *p* < 0.01, $\eta_{p}^{2}$= 0.75, see Table 2 and Figure 2C]. For both groups, the congruency effect is smaller for the motion- than for the arrow Flanker-Task [Stimulus × Congruency: *F*_(1,40)_ = 57.8, *p* < 0.01, $\eta_{p}^{2}$= 0.59] due to lower accuracy already in the congruent RDP compared to the static arrowhead configuration.

While the groups’ error rates do not differ overall [Group: *F*_(1,40)_ = 2.5, *p* = 0.12, $\eta_{p}^{2}$= 0.06], children with ADHD produced smaller congruency effects in both tasks than control children [23% vs. 16% Δ error-rates in the arrow Flanker-Task, 11% vs. 5% in the motion Flanker-Task, Congruency × Group: *F*_(1,40)_ = 5.7, *p* = 0.02, $\eta_{p}^{2}$ = 0.13, this may be due to the performance feedback, see the discussion of performance data]. However, overall accuracy in processing RDPs was especially diminished in children with ADHD [Stimulus × Group: *F*_(1,40)_ = 6.1, *p* = 0.02, $\eta_{p}^{2}$= 0.13].

### Brain Electrical Activity

#### Stimulus-Locked Cognitive Control (N2-Enhancement)

**FIGURE 3:**
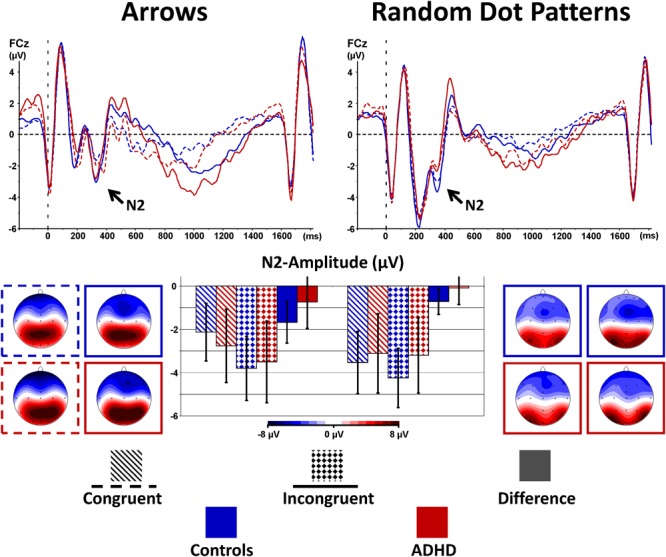
Stimulus-locked N2-enhancement. Cognitive control tapped in incongruent (solid waveforms with target-onset at t = 0 and checkered bars giving confidence intervals with p = 0.05) compared to congruent (dashed waveforms and dashed bars) stimuli was associated with enhanced N2-amplitudes particularly in controls. This N2-enhancement was lower in the RDP Flanker-Task, and diminished in boys with ADHD.

The stimulus-locked ERPs show fronto-central negativity, peaking around 200–400 ms after onset of the stimulus at FCz, where the N2 peaks and the N2 enhancement were most pronounced. Incongruent stimuli evoke enhanced N2 amplitudes compared to congruent ones for both groups and both tasks [Congruency: *F*_(1,40)_ = 11.9, *p* < 0.01, $\eta_{p}^{2}$= 0.23, see Table 3 and Figure 3], but this effect was as a trend smaller for RDPs [Stimulus × Congruency: *F*_(1,40)_ = 3.4, *p* = 0.07, $\eta_{p}^{2}$= 0.08]. There was further a trend for lower N2-enhancement in the ADHD-group [Congruency × Group: *F*_(1,40)_ = 2.8, *p* = 0.10, $\eta_{p}^{2}$= 0.07], particularly at Fz and FCz than Cz [Congruency × Electrode × Group: *F*_(2,80)_= 3.3, 𝜀 = 0.61^∗∗^, *p* = 0.07, $\eta_{p}^{2}$= 0.08], but irrespectively of stimuli used [Stimulus × Congruency × Group: *F*_(1,40)_ = 0.1, *p* = 0.72, $\eta_{p}^{2}$< 0.01].

**Table 3:** Stimulus-locked electrophysiological data of flanker effects with statistics.

	Controls	ADHS	ANOVA			
Stimulus-locked N2	Mean (*SD*)	Mean (*SD*)	Test	*F*_(1,40)_	*p*	$\eta_{p}^{2}$
**Latency at FCz (ms):**
**Arrow flanker task**						
Congruent correct	346 (32)	357 (28)	S G **C** S × G C × G S × C S × C × G	0.5 0.1 **8.0** 0.2 1.6 0.2 0.7	– - **^∗∗^** - - - -	0.01 0.01 **0.17** 0.01 0.04 0.01 0.02
Incongruent correct	342 (33)	341 (31)				
**Motion flanker task**						
Congruent correct	345 (43)	343 (47)				
Incongruent correct	339 (49)	336 (30)				
**Amplitude (μV):**
**Arrow flanker task**						
Congruent correct						
Fz	–4.0 (3.5)	–5.2 (3.9)	S G **C** **Site** S × G **C × G** **S × C** S × C × G G × Site **S × Site** S × G × Site C × Site **C × G × Site** S × C × Site S × C × G × Site	1.5 0.1 **11.9** **35.6** 1.4 2.8 3.4 0.1 0.6 17.6 0.1 1.3 3.3 0.1 0.5	– - **^∗∗^** **^∗∗^** - **+** **+** - - **^∗∗^** - - **+** - -	0.04 0.04 **0.23** **0.47** 0.03 **0.07** **0.08** 0.01 0.02 **0.31** 0.01 0.03 **0.08** 0.01 0.01
FCz	–2.9 (4.2)	–3.2 (4.1)				
Cz	0.5 (3.7)	0.1 (3.5)				
Incongruent correct						
Fz	–5.7 (3.9)	–5.7 (5.5)				
FCz	–5.2 (4.6)	–4.1 (4.5)				
Cz	–0.5 (3.9)	–0.7 (4.5)				
**Motion flanker task**						
Congruent correct						
Fz	–3.9 (3.6)	–4.1 (4.4)				
FCz	–4.3 (3.5)	–3.5 (4.8)				
Cz	–2.4 (3.2)	–1.7 (5.1)				
Incongruent correct						
Fz	–4.9 (3.6)	–3.7 (5.0)				
FCz	–5.4 (3.5)	–3.6 (4.7)				
Cz	–2.4 (3.4)	–2.3 (4.9)				
**Number of accepted sweeps**						
**Arrow flanker task**						
Congruent correct	163(37.2)	125 (49.8)	S	3.2	+	0.07
Incongruent correct	118 (32.3)	91 (39.3)	**C**	**108.6**	^∗∗^	**0.73**
**Arrow flanker task**			**C^∗^G**	3.0	+	0.07
Congruent correct	148 (46)	99 (53.6)	**S × C**	68.6	**^∗∗^**	**0.63**
Incongruent correct	128 (39.1)	86 (44.0)	**G**	**10.2**	**^∗∗^**	**0.20**

****Left side:****Stimulus-locked electrophysiological data of both groups for N2 components of correct responded congruent and incongruent configurations for Arrow flanker task and Motion flanker task: latencies in ms and amplitudes in μV at electrodes Fz, FCz, and Cz.***Right side:** Statistics performed by analysis of variance (ANOVA): S, stimulus; G, group; C, congruency, Site electrodes Fz, FCz, and Cz (df = 2, 80). ^∗∗^p < 0.01 and +p < 0.10. Bold lines indicate at least medium effects with at least *p* < 0.10.*

#### Response Processing (Ne/Nc and Pe/Pc)

Typically, the responses to attention-demanding tasks are more error-prone in children with ADHD. We therefore analyzed error processing components of the ERP, generally embodied by a negative deflection peaking at fronto-central sites approximately 40–120 ms after the error response (error negativity, Ne) that is followed by a more parietal positive deflection (error positivity, Pe) within 200–500 ms after the response (Falkenstein et al., 1990; Gehring et al., 1993). Both Ne and Pe were compared with the activities evoked by correct responses Nc and Pe, respectively.

**FIGURE 4:**
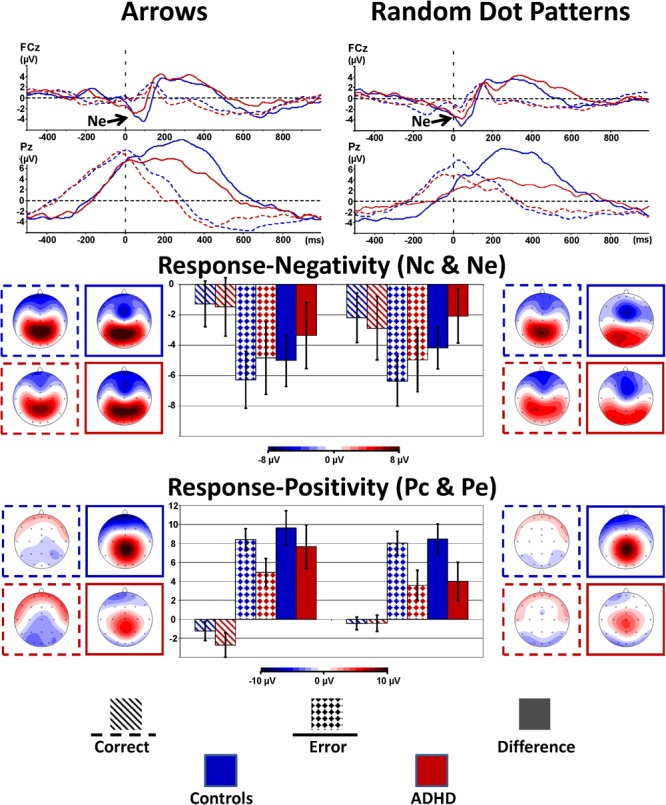
Response-locked error processing. Response-locked activity to correct responses (dashed) compared to errors (solid lines, squared bars giving confidence intervals with *p* = 0.05) showed elevated negativities (Ne, fronto-central maximum) and positivities (Pe, centro-parietal maximum) to errors; the respective differences to activity evoked by correct responses were diminished in ADHD.

Figure 4 shows the response-locked grand average waveforms and activity maps as well as *post hoc* comparisons with confidence intervals (*p* = 0.05). The error negativity (measured as the peak within 150 ms following the response) is most pronounced at the fronto-central electrode FCz, whereas the adjacent error positivity (quantified as mean amplitude 200–500 ms following the response) is maximal at the centro-parietal electrode Pz.

The amplitude for the early response negativity is overall similar in static arrowheads and RDPs [Stimulus: *F*_(2,80)_ = 2.2, *p* = 0.15, $\eta_{p}^{2}$= 0.05], and larger following errors than after correct responses [Error: *F*_(1,40)_ = 51.0, *p* < 0.01, $\eta_{p}^{2}$= 0.56], similarly in Arrowheads and RDPs [Stimulus: *F*_(1,40)_ = 2.2, *p* = 0.15, $\eta_{p}^{2}$= 0.05 and Stimulus^∗^Error: *F*_(1,40)_ = 2.1, *p* = 0.15, $\eta_{p}^{2}$= 0.05]. The error-specific increase in this early negativity was as a trend larger in Controls than children with ADHD [Error^∗^Group: *F*_(1,40)_ = 3.3, *p* = 0.08, $\eta_{p}^{2}$= 0.08 and Group: *F*_(1,40)_ = 0.2, *p* = 0.66, $\eta_{p}^{2}$< 0.01, see Figure 4 and Table 4 for further details]. In addition, we explored the Nc in more detail, as it may reflect task difficulty or ambiguity differences between static and motion stimuli, and found elevated Nc amplitudes in the RDP Flanker-Task, similar in ADHD and Controls [*F*(1,40) = 7.7, *p* < 0.01, $\eta_{p}^{2}$= 0.16].

**Table 4:** Response-locked electrophysiological data of error processing with statistics.

	Controls *N* = 26	ADHD *N* = 16	ANOVA			
Response-locked	Mean (*SD*)	Mean (*SD*)	Test	*F*_(1,40)_	p	$\eta_{p}^{2}$
**Response negativity: latency at FCz (ms)**
**Arrow flanker task**			**S**	**22.3**	**^∗∗^**	**0.36**
Nc	46 (24)	46 (33)	G	2.8	–	0.07
Ne	74 (37)	51 (39)	S × G	0.1	–	<0.01
**Motion flanker task**			**E**	**4.7**	**^∗^**	**0.11**
Nc	34 (22)	36 (14)	**E × G**	**5.9**	**^∗^**	**0.13**
Ne	49 (26)	29 (32)	S × E	2.5	–	0.06
			S × E × G	<0.01	–	<0.01
**Response negativity: amplitude at FCz (μV):**
**Arrow flanker task**			S	2.2	–	0.05
Nc	–1.3 (4.2)	–1.5 (3.1)	G	0.2	–	<0.01
			S × G	0.1	–	<0.01
Ne	–6.3 (4.6)	–4.8 (5.1)	**E**	**51.0**	**^∗∗^**	**0.56**
			**E × G**	**3.3**	**+**	**0.08**
Δ (Ne-Nc)	–5.0 (4.1)	–3.4 (4.7)	S × E	2.1	–	0.05
**Motion flanker task**			S × E × G	0.1	–	<0.01
Nc	–2.2 (4.4)	–2.9 (3.7)				
Ne	–6.4 (4.5)	–4.8 (5.1)				
Δ (Ne-Nc)	–4.2 (3.4)	–2.1 (3.6)				
**Response positivity: mean amplitude +200 to +500 ms at Cz and Pz (μV):**
**Arrow flanker task**						
Pc						
Cz	–1.1 (2.8)	–2.6 (3.0)	S **G** S × G **E** **S × E** **E × G** **E × S × G** **Site** **E × Site** **E × G × Site**	2.4 **28.9** <1 **166.5** **11.1** **7.8** **2.9** **4.0** **4.7** **2.8**	– **^∗∗^** - **^∗∗^** **^∗∗^** **^∗∗^** **+** **+** **^∗^** **+**	0.06 **0.42** <0.01 **0.81** **0.22** **0.16** **0.07** **0.09** **0.11** **0.07**
Pz	–1.4 (2.7)	–2.8 (3.4)				
Pe						
Cz	7.1 (3.7)	4.2 (2.5)				
Pz	9.7 (3.5)	5.7 (4.0)				
**Motion flanker task**						
Pc						
Cz	–0.7 (2.6)	–0.8 (1.9)				
Pz	–0.2 (2.1)	0.0 (2.4)				
Pe						
Cz	7.2 (3.9)	3.8 (3.4)				
Pz	8.9 (3.0)	3.4 (3.7)				
**Number of accepted sweeps**						
**Arrow flanker task**						
Correct response	120 (31.9)	95 (38.7)				
Error	53 (20.4)	47 (14.9)	**C**	**108.8**	**^∗∗^**	**0.73**
**Motion flanker task**			**S × C × G**	**4.0**	**^∗^**	**0.09**
Correct response	130 (37.8)	90 (41.8)	**G**	**8.6**	**^∗^**	**0.18**
Error	40 (12.5)	47 (14.5)				

****Left side:****Response-locked electrophysiological data of both groups for incorrect responded incongruent trials for the Arrow- and the Motion flanker task: Response negativity to correct responses (Nc) and errors (Ne) with latencies in ms and amplitudes in μV at electrodes FCz, and response positivity (Pc and Pee) with amplitudes (μV) at electrodes Cz and Pz.****Right side:****Statistics performed by analysis of variance (ANOVA): S, stimulus; G, group; E, error; Site electrodes Cz and Pz. ^∗∗^p < 0.01, ^∗^p < 0.05, +p < 0.1.* Bold lines indicate at least medium effects with at least *p* < 0.10.*

The following adjacent response positivity was larger following errors than correct responses [Error: *F*_(1,40)_ = 166.5, *p* < 0.01, $\eta_{p}^{2}$= 0.81], which was more pronounced in Controls than children with ADHD [Error × Group: *F*_(1,40)_ = 7.8, *p* < 0.01, $\eta_{p}^{2}$= 0.16]. These effects were smaller in RDPs than Arrows [Stimulus × Error: *F*_(1,40)_ = 11.1, *p* < 0.01, $\eta_{p}^{2}$= 0.22], which was as a trend more pronounced in ADHD [Stimulus^∗^Error^∗^Group: *F*_(1,40)_ = 2.9, *p* = 0.09, $\eta_{p}^{2}$= 0.07, see Figure 4 and Table 4]. In addition, this “Pe-Enhancement” was larger at the more posterior site Pz than Cz [Site: *F*_(1,40)_ = 4.0, *p* = 0.05, $\eta_{p}^{2}$= 0.09, Error × Site: *F*_(1,40)_ = 4.7, *p* = 0.04, $\eta_{p}^{2}$= 0.11, Error × Site × Group: *F*_(1,40)_ = 2.8, *p* = 0.10, $\eta_{p}^{2}$= 0.07, Error × Stimulus × Site: *F*_(1,40)_ = 18.2, *p* < 0.01, $\eta_{p}^{2}$= 0.31].

#### Total Alpha Power During Flanker-Task Performance

**FIGURE 5:**
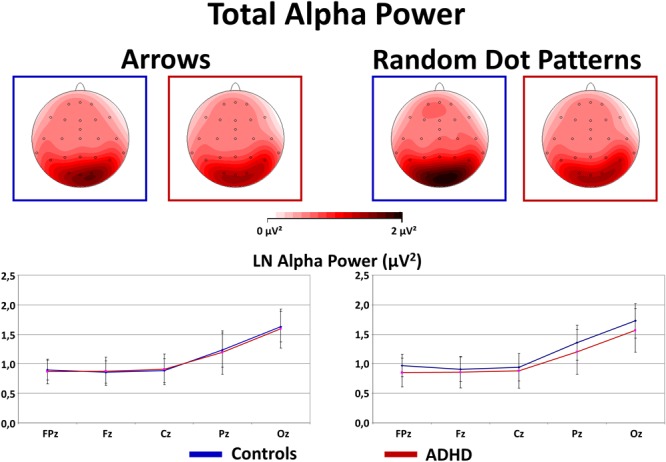
Total alpha power during flanker-task performance. This figure gives total power in the Alpha (8–13 Hz) frequency band during Flanker-Task performance. As expected, Alpha power is elevated at occipital sites (see topography maps above and confidence intervals with *p* = 0.05 of marginal means from midline electrodes below). Contrary to expectations, Controls show elevated Alpha activity when processing RDPs compared to static arrowheads, which was for RDPs also higher than in children with ADHD.

As expected, Alpha power was strongest over occipital and parietal sites [Site *F*_(4,160)_ = 53.2, *p* < 0.01, $\eta_{p}^{2}$= 0.57]. In contrast to our expectations, an interaction Stimulus × Group [*F*_(1,40)_ = 4.0, *p* = 0.05, $\eta_{p}^{2}$= 0.09] was driven by overall higher Alpha power in Controls compared to ADHD when processing RDPs (but this effect reached no significance at any particular electrode, see Figure 5 for confidence intervals with *p* = 0.05), while no group differences emerged when processing static arrowheads (see Figure 5).

## Discussion

Previous studies have documented cognitive control deficits in ADHD with Flanker- or Go/Nogo tasks based on processing static visual forms and after performance errors (Jonkman et al., 2007; Albrecht et al., 2008; McLoughlin et al., 2009; Mullane et al., 2009), but very little is known about cognitive control during visual motion processing, although this ability is a critical core ability of primate visual systems. Here, we compared performance and brain electrical activity during Flanker-Tasks employing classical static stimuli (vertical arrangements of arrowheads) on the one hand and visual motion stimuli (moving RDPs) on the other hand.

### Performance

Processing incongruent Flankers requires elevated cognitive control, which leads to slower and more error-prone responses to incongruent stimuli in both Flanker-Tasks. Congruency effects for reaction times and accuracy are significantly smaller in the motion Flanker-Task compared to the static arrowhead task for ADHD and control children. The smaller motion congruency effects are driven by prolonged reaction times and higher error rates already in the congruent motion condition, and may thus indicate general performance problems when processing motion rather than specific impairments in cognitive control. In addition, we detected elevated reaction time variability and thus more heterogeneous performance in the motion Flanker-Task. These findings of diminished motion processing performance may be explained by an immature developmental status of the dorsal visual processing stream in childhood, with full maturation probably not reached until adolescence (Bucher et al., 2006; Langrová et al., 2006; Booth et al., 2007; Klaver et al., 2008). Correspondingly, in a recent study with adults, differences in performance between form- and motion Flanker-Task were absent (Lange-Malecki and Treue, 2012), which suggests that the development of visual motion processing capabilities is still ongoing in our subjects’ age range. With increasing age the children’s ability to respond more automatically due to practice enhances [see also the reverse Stroop effect (MacLeod, 1991)], and response organization, especially for moving stimuli (Li et al., 2008), improves.

Our observation of diminished Flanker-Task performance in children with ADHD compared to controls matches previous studies. However, significantly smaller congruency effects of the ADHD group particularly in accuracy contrasts to the larger congruency effects in ADHD reported by other studies (Johnson et al., 2008; Mullane et al., 2009). This may be the consequence of the performance feedback we employed to ensure similar total error rates in all children, making response times more reliable by avoiding group differences in speed-accuracy trade-off which can modulate error processing and cognitive control (Falkenstein et al., 2000). It is well known that patients with ADHD make generally more errors than typically developed children, but in the current tasks no overall group difference was found, suggesting that the performance feedback had the intended effect of keeping overall error rates similar across groups. Probably as a consequence, children with ADHD showed significantly diminished congruency-effects for accuracy, driven by enhanced error rates in congruent trials, with the strongest effect found in the motion flanker task (see Figure 2 and Table 2).

Taken together, both Flanker-Tasks using classic static arrowheads and moving RDPs yielded expected performance modulations when cognitive control is required. In addition, motion processing *per se* is especially difficult for children, as the dorsal visual stream, responsible for this visual ability, may undergo maturation until adolescence. Children with ADHD show impairments in both tasks, but motion processing irrespectively of cognitive control is particularly challenging for patients.

### Brain Electrical Activity

Cognitive Control demands do also lead to modulations in brain electrical activity. The current data revealed a trend for lower N2-enhancement due to stimulus incongruency similarly for moving and static stimuli, but the detected medium effect is slightly smaller than the case-control difference in our previous familiality study (Albrecht et al., 2008). Besides limited statistical power, the children with ADHD tested in the current study show less severe symptoms, which further limit ADHD-effects in the current study.

Importantly, N2-enhancement was (as a trend) smaller in the motion Flanker-Task, which was particularly driven by larger N2-amplitudes already in the congruent condition, a similar pattern of results as detected for reaction times, again suggesting some ambiguity when processing visual motion which potentially taps into cognitive control already in the congruent condition. Our findings regarding brain electrical activity associated with cognitive control complement and differentiate findings from performance data: we detected N2-enhancement and error-negativity and -positivity, indicating – even if the maturation of the visual motion processing system is still ongoing – that cognitive control may be diminished in children with ADHD not only when processing static visual forms, but also when processing visual motion in Flanker-Tasks.

Additional evidence for motion processing deficits in ADHD comes from a recent study with biological motion. In a simple identification task where moving walkers had to be identified from scrambled moving random dots, children with ADHD did not differ in identification rate from typically developed children, but they showed a lowered vN1 (labeled as N200) with a more diffuse activation in occipital-temporal regions (Kröger et al., 2014). Taken together, children with ADHD show difficulties during early neuronal processing of more complex “biological” visual motion.

In the response-locked ERP, we observed (as a trend, but toward medium effect size, in line with the literature) smaller error negativity- and (significantly) lower positivity- amplitudes (assessed as difference amplitudes with respect to correct response Nc and Pc) for children with ADHD compared to controls. These findings may indicate reduced conflict between error and correct response, and also diminished motivational error assessment particularly in ADHD. But things tend to be more complicated, as the RDP Flanker-Task evoked enhanced early negativity to correct responses (Nc) similarly in both groups, and diminished Pe difference amplitude particularly in ADHD, which may be explained by higher error-rates leading to less error salience in that condition. Both enhanced Nc and lower Pe (difference) amplitudes may thus indicate some more ambiguity in responding to RDPs compared to static arrowheads. This is at least regarding Performance in stark contrast to our previous study with healthy adults that showed very similar Flanker-Task performance for both static arrowheads and RDPs. We may conclude so far that the motion processing Flanker-Task is more difficult for children than adults, which may be a consequence of ongoing maturation of the dorsal processing stream with full development probably not reached until adolescence (Bucher et al., 2006; Klaver et al., 2008). This may also explain heterogeneity in motion recognition performance reviewed by Hadad et al. (2015) that give estimates when performance become adult-like between 3 and 16 years, probably pending on demand and degree of feature integration (Hadad et al., 2011).

Assessment of absolute Alpha activity during task performance yielded elevated power in Controls during RDP processing, suggesting that control children had lower activation during Flanker-Task motion processing - or payed less attention to RDPs. Elevated alpha activity during task performance in controls has also been reported by Loo et al. (2009) from a Continuous Performance Test (CPT), and the authors interpreted their findings in terms of lower arousal/activation in Controls that may perform such a rather easy sustained attention task (that had ceiling effects in performance data) more efficiently than children with ADHD. However, such an explanation does not hold for the current motion Flanker-Task, as performance in the RDP Flanker-Task has dropped compared to the version using static arrowheads. One may speculate whether elevated alpha activity with sources in the visual areas (as likely the case in the current data) may indicate a suppression mechanism for task-irrelevant visual stimuli as elaborated by Klimesch (2012). With regard to the CPT data reported by (Loo et al., 2009), one may speculate whether the detected elevated alpha activity in Controls may come from event-related alpha synchronization generated by active suppression of processing distractors (that are in the CPT much more frequent than targets that require active responding). Further studies may differentiate attention deficits in patients with ADHD regarding their abilities of selecting relevant from suppressing distracting (or to them even uninteresting?) information.

### Limitations

The limited sample size of 26 Controls and 16 children with ADHD, and an alpha error set to a conventional 5% allows the detection of large phenotype effects (*d* ≥ 0.8) (Cohen, 1988) with a power of 80%. The consideration of trends with α < 0.10 would allow the detection of medium sized effects (*d* ≥ 0.5) with a power of only 60%. As a consequence, a number of important effects may remain undiscovered in the current study, whilst trends need to be considered with care. However, the current study aims extending previous perspectives on Cognitive control in ADHD, so we explicitly test hypotheses which warrant the current approach.

Another difficulty, especially with visually evoked potentials, is the consideration of eye movements throughout the task, as eye movements induce artifacts into the recorded brain activity and may indicate that something interrupted the task performance. For the current study, a consequent rejection of contaminated trials would lead to an additional dropout of 30% of the sample. In a supplementary analysis we compared the impact of ocular correction versus blink rejection in the remaining sample. While the applied regression-based ocular correction procedure may effectively eliminate ocular artifacts, it also reduces activity at fronto-polar sites. However, effects on the fronto-central electrodes included in the current analyses were rather small and are unlikely to have compromised the reported results.

Another difficulty with the current design is the fixed flanker-target asynchrony which led to an overlap of the ERP components evoked by target and flankers as well as responses. While the latter may be ameliorated by techniques like Adjar (Woldorff, 1993) or RIDE (Ouyang et al., 2015), the overlap of activity evoked by Flankers and Target remains as a difficulty. As this study aims compatibility with previous works we adopted in the current study a classical approach that is directly comparable to previous findings (Albrecht et al., 2008; McLoughlin et al., 2009).

Assessments of medication effects were beyond the scope of this study. While discontinuing medication with stimulants for 48 h prior testing is a good standard in research with patients, the ongoing use of medication using norepinephrine reuptake inhibitors like Atomoxetine© may lead to underestimated ADHD effects.

In sum, although some weaknesses of the current study cannot be ruled out, these obstacles will not compromise the validity of the reported results.

## Conclusion

The current study confirms medium effect-sized deficits in cognitive control in children with ADHD compared to typically developing controls when processing static visual forms in a Flanker-Task regarding performance (reaction time and -variability and error-rate) as well as brain electrical activity (N2-Enhancement due to stimulus incongruency and in elevated Ne and Pe amplitudes after errors). This not so surprising finding was extended with data from a parallel form of the Flanker-Task using moving RDPs that may capture automatic “bottom-up” attention and may thus lead to more severe deficits in ADHD.

The current findings revealed that processing moving RDPs was more challenging for both groups of children, leading to slower RTs and higher error-rates already in the congruent condition and generally higher RT-SD, which was paralleled by elevated error processing deficits, probably as a consequence of immature visual motion processing capabilities at this age. In contrast to our hypothesis, deficits in cognitive control were similarly present when processing static and motion Flanker-Tasks, indicating a higher order cognitive control deficit in ADHD.

## Author Contributions

BL-M conceived the study, organized data collection, and drafted the manuscript. ST conceived the study and drafted the manuscript. AR conceived the study and drafted the manuscript. BA conceived the study, organized data collection, performed analyses, and drafted the manuscript.

## Conflict of Interest Statement

The authors declare that the research was conducted in the absence of any commercial or financial relationships that could be construed as a potential conflict of interest.
